# Cisplatin enhances cell stiffness and decreases invasiveness rate in prostate cancer cells by actin accumulation

**DOI:** 10.1038/s41598-018-38199-7

**Published:** 2019-02-07

**Authors:** Martina Raudenska, Monika Kratochvilova, Tomas Vicar, Jaromir Gumulec, Jan Balvan, Hana Polanska, Jan Pribyl, Michal Masarik

**Affiliations:** 1 Department of Physiology, Faculty of Medicine, Masaryk University/Kamenice 5, CZ-625 00, Brno, Czech Republic; 2 Department of Pathological Physiology, Faculty of Medicine, Masaryk University/Kamenice 5, CZ-625 00 Brno, Czech Republic; 3 Department of Biomedical Engineering, Faculty of Electrical Engineering and Communication, Brno University of Technology, Technicka 3058/10, CZ-616 00 Brno, Czech Republic; 4 Central European Institute of Technology, Brno University of Technology, Technicka 3058/10, CZ-616 00 Brno, Czech Republic; 5 Central European Institute of Technology, Masaryk University/Kamenice 5, CZ-625 00 Brno, Czech Republic

**Keywords:** Cellular motility, Cell invasion

## Abstract

We focused on the biomechanical and morphological characteristics of prostate cancer cells and their changes resulting from the effect of docetaxel, cisplatin, and long-term zinc supplementation. Cell population surviving the treatment was characterized as follows: cell stiffness was assessed by atomic force microscopy, cell motility and invasion capacity were determined by colony forming assay, wound healing assay, coherence-controlled holographic microscopy, and real-time cell analysis. Cells of metastatic origin exhibited lower height than cells derived from the primary tumour. Cell dry mass and *CAV1* gene expression followed similar trends as cell stiffness. Docetaxel- and cisplatin-surviving cells had higher stiffness, and decreased motility and invasive potential as compared to non-treated cells. This effect was not observed in zinc(II)-treated cells. We presume that cell stiffness changes may represent an important overlooked effect of cisplatin-based anti-cancer drugs. Atomic force microscopy and confocal microscopy data images used in our study are available for download in the Zenodo repository (https://zenodo.org/, Digital Object Identifiers:10.5281/zenodo.1494935).

## Introduction

Atomic force microscopy (AFM) is a three-dimensional high-resolution topographic technique suitable for biological applications in native conditions^1^ with the ability to measure cantilever probe bending with an extremely high precision^2^. Moreover, AFM emerged as a powerful tool to obtain biomechanical properties of biological samples including biomolecules and cells^1,3–6^. The method of nanomechanical mapping of cell surfaces is based on works published by Nikolaev and Thomas^7,8^.

It was shown that cell stiffness determined by AFM can be used as a marker for cancer progression and metastatic potential^9–11^. Different cancer types feature distinct cell stiffness^12^ and a connection between attenuated cell stiffness and increased invasion capacity was also observed^13^. Furthermore, cytoskeletal architecture changes induced by stress (anti-cancer drugs or fluid shear stress in the circulatory system during metastatic processes) were shown to influence biomechanical features of cancer cells significantly^4,14,15^. Since the cellular bio-mechanical characteristics including cell stiffness are very important for cell motility^9^, changes in the cytoskeletal architecture and consequent changes in the cell stiffness, cell dry mass, and motility could represent important secondary effects of many cytostatic drugs.

We studied the effect of two widely used anticancer drugs docetaxel and cisplatin on a panel of prostate cancer cell lines by using AFM, quantitative phase imaging and assays analyzing migratory and invasiveness potentials. Furthermore, the effect of zinc supplementation on the biomechanical characteristics of prostate cancer cells was also tested because zinc(II) ions play a key role in the prostate gland metabolism and contribute to the number of biological processes such as apoptosis, signal transduction and cell invasiveness^16–18^. Docetaxel is a second-generation taxane derived from the needles of *Taxus baccata*. The primary mechanism of action for docetaxel is to promote and stabilize microtubulin assembly, thereby blocking microtubule dynamics. Consequences include impairment of mitotic progression, cell cycle arrest, and inhibition of cell proliferation^19^. Furthermore, some studies indicate that DNA is not the only cellular target for cisplatin, but that it may also affect cytoskeleton^20,21^. These additional interactions could enhance the anti-proliferative effect and contribute to the anti-cancer effects of cisplatin such as inhibition of growth and migration. An important aim of this study was to reveal changes in the cell stiffness after treatment (zinc, docetaxel, cisplatin) and to assess the effect of this changed cell stiffness on cell invasiveness and migration of prostate cancer cells in different stages of cancer disease progression. The second question we wanted to answer was whether the expression of *CAV1* gene in prostate cancer cells reflects their bio-mechanical phenotypes because Cav1 has been recently linked to cell stiffness through the regulation of actin remodelling and focal adhesions^22,23^.

## Methods

### Chemical and biochemical reagents

RPMI-1640 medium, Ham’s F12 medium, fetal bovine serum (FBS) (mycoplasma-free), penicillin/streptomycin, and trypsin were purchased from Sigma Aldrich Co. (St. Louis, MO, USA). Phosphate buffered saline PBS was purchased from Invitrogen Corp. (Carlsbad, CA, USA). Ethylenediaminetetraacetic acid (EDTA), zinc(II) sulphate (BioReagent grade, suitable for cell cultures) and all other chemicals of ACS purity including docetaxel were purchased from Sigma Aldrich Co. (St. Louis, MO, USA) unless noted otherwise.

### Cell cultures

Four human prostatic cell lines were used in this study. The PNT1A human cell line is derived from normal adult prostatic epithelial cells immortalized by transfection with a plasmid containing SV40 genome with defective replication origin. The primary culture was obtained from the normal prostatic tissue of a 35-year old male *post-mortem*. PNT1A is PTEN positive non-tumorigenic epithelial cell line^24^. PNT1A cells harbour wild-type p53. However, SV40 induced T-antigen expression inhibits the activity of p53^25,26^. This cell line had lost the expression of androgen receptor (AR) and prostate-specific antigen (PSA)^27,28^. 22Rv1 is the human prostate carcinoma epithelial cell line derived from a xenograft serially propagated in mice after castration. The cell line expresses prostate specific antigen (PSA). Growth is weakly stimulated by dihydroxytestosterone and lysates are immunoreactive with AR antibody. 22Rv1 is PTEN and p53 positive^29,30^. The PC-3 human epithelial cell line was established from a 4-grade prostatic adenocarcinoma, androgen-independent and unresponsive metastatic site in the bone. PC-3 is PTEN-, AR-, PSA-, and p53-negative^26,28,29^. The LNCaP cell line was established from a lymph node metastasis of the hormone-refractory patient and contains a mutation in the AR gene. This mutation creates a promiscuous AR that can bind to different types of steroids. LNCaP are AR-positive, PSA-positive, PTEN-negative and harbor wild-type p53^28,30^. All cell lines used in this study were purchased from HPA Culture Collections (Salisbury, UK).

### Cell cultivation

The PNT1A, LNCaP, and 22Rv1 cells were cultured in the RPMI-1640 medium with 10% FBS. The PC-3 cells were cultured in the Ham’s F12 medium with 10% FBS. Both media were supplemented with antibiotics (penicillin 100 U/ml and streptomycin 0.1 mg/ml). The cells were maintained at 37 °C in the humidified (60%) incubator with 5% CO_2_ (Sanyo, Japan).

### MTT viability assay

The MTT assay was used to determine the cell viability. After a passage, the suspension of cells in growth media was diluted to a concentration of 2 000–10 000 cells/200 ul and transferred into 96-well plate. On each plate, positive and negative control was carried out. The plates were incubated for 2 days at 37 °C to ensure cell adhesion. Docetaxel and cisplatin were added in fresh media at increasing concentrations (0–400 nM for docetaxel and 0–150 μmol/l for cisplatin). The plates with the treatment were incubated for 24 h. Subsequently, the medium was changed to a fresh medium with MTT (4:1, MTT 5 mg/ml in PBS) and incubated for 4 h in the incubator in the dark. DMSO was used to dissolve MTT – formazan crystals and absorbance was measured at 570 nm (VersaMax microplate reader, USA).

### Cisplatin and docetaxel treatment of cell cultures

Cells confluent up to 50–60% were washed with a FBS-free medium and treated with a fresh medium with FBS and required antineoplastic drug concentration (IC50 concentration for the particular cell line). The cells were treated with 93 µM (PC-3), 38 µM (PNT1A), and 24 µM (22Rv1) of cisplatin (Sigma-Aldrich, St. Louis, Missouri), respectively. IC50 concentrations used for treatment with docetaxel (Sigma-Aldrich, St. Louis, Missouri) were 200 nM for PC-3, 70 nM for PNT1A, and 150 nM for 22Rv1. The cells were cultivated under these conditions for 24 h. Subsequently, the cells were washed with an FBS-free medium and treated with a fresh medium with FBS. Non-viable cells were washed out and the viability of remaining cells was checked by microscopy. AFM, coherence-controlled holographic microscopy, invasion assay, colony forming assay and wound healing assay followed.

### Long-term zinc (II) treatment of cell cultures

Cells were cultivated in the constant presence of zinc(II) ions. Concentrations of zinc(II) sulphate in the medium were increased gradually by small changes of 25 or 50 µM. The cells were cultivated at each concentration no less than one week before harvesting and their viability was checked before adding more zinc. This process was used to select zinc resistant cells naturally and to ensure better accumulation of zinc within the cells (accumulation of zinc is usually poor during the short-term treatment of prostate cancer cells)^18^. Total time of the cultivation of cell lines in the zinc(II)-containing media exceeded one year. Resulting concentrations of zinc(II) in the media (IC50 for the particular cell line) were 50 µM for the PC-3 cell line, 150 µM for the PNT1A cell line, and 400 µM for the 22Rv1 cell line. The concentrations of zinc(II) in the media and FBS were taken into account. The cells were washed with an FBS-free medium and treated with a fresh medium with FBS. AFM, coherence-controlled holographic microscopy, invasion assay, colony forming assay and wound healing assay followed.

### RNA isolation, cDNA preparation

The cultivation medium was removed and the cells were washed with PBS and trypsinized. TriPure Isolation Reagent (Roche, Basel, Switzerland) was used for RNA isolation. RNA samples without reverse transcription were used as negative control for qRT-PCR to exclude DNA contamination. The isolated RNA was used for the cDNA synthesis. RNA (1000 ng) was transcribed using the transcriptor first strand cDNA synthesis kit (Roche, Switzerland) according to manufacturer’s instructions. The cDNA (20 μl) prepared from the total RNA was diluted with RNase free water to 100 μl and the amount of 5 μl was analyzed directly.

### Quantitative real-time polymerase chain reaction (qRT-PCR)

The qRT-PCR was performed using TaqMan gene expression assays with the LightCycler®480 II System (Roche, Basel, Switzerland). The amplified DNA was analyzed by the comparative Ct method using β-actin as a reference. The primer and probe sets for *ACTB* (assay ID: Hs99999903_m1), and CAV1 (assay ID: Hs00971716_m1) were selected from the TaqMan gene expression assays (Life Technologies, USA). The qRT-PCR was performed under following amplification conditions: total volume of 20 μl, initial incubation at 50 °C/2 min followed by denaturation at 95 °C/10 min, then 45 cycles at 95 °C/ 15 sec and at 60 °C/1 min.

### Actin and tubulin staining

β-tubulin was labeled with anti- β tubulin antibody [EPR1330] (ab108342) at a working dilution of 1/300. The secondary antibody used was Alexa Fluor® 555 donkey anti-rabbit (ab150074) at a dilution of 1/1000. Actin was labeled with Alexa Fluor™ 488 Phalloidin (A12379, Invitrogen); 1 unit per slide. For mounting Duolink® *In Situ* Mounting Medium with DAPI (DUO82040) was used. The cells were fixed in 3.7% paraformaldehyde and permeabilized using 0.1% Triton X-100.

### Confocal microscopy

The microscopy of samples was performed at the Institute of Biophysics, Czech Academy of Sciences, Brno, Czech Republic. Leica DM RXA microscope (equipped with DMSTC motorized stage, Piezzo z-movement, MicroMax CCD camera, CSU-10 confocal unit and 488, 562, and 714 nm laser diodes with AOTF) was used for acquiring detailed cell images (100 × oil immersion Plan Fluotar lens, NA 1.3). Total 50 Z slices was captured with Z step size 0.3 μm.

### AFM measurements

We used the bioAFM microscope JPK NanoWizard 3 (JPK, Berlin, Germany) placed on the inverted optical microscope Olympus IX-81 (Olympus, Tokyo, Japan) equipped with the fluorescence and confocal module, thus allowing a combined experiment (AFM-optical combined images). The maximal scanning range of the AFM microscope in X-Y-Z range was 100-100-15 µm. The typical approach/retract settings were identical with a 15 μm extend/retract length, Setpoint value of 1 nN, a pixel rate of 2048 Hz and a speed of 30 µm/s. The system operated under closed-loop control. After reaching the selected contact force, the cantilever was retracted. The retraction length of 15 μm was sufficient to overcome any adhesion between the tip and the sample and to make sure that the cantilever had been completely retracted from the sample surface. Force-distance (FD) curve was recorded at each point of the cantilever approach/retract movement. AFM measurements were obtained at 37 °C (Petri dish heater, JPK) with force measurements recorded at a pulling speed of 30 µm/s (extension time 0.5 sec).

The Young’s modulus (E) was calculated by fitting the Hertzian-Sneddon model on the FD curves measured as force maps (64 × 64 points) of the region containing either a single cell or multiple cells. JPK data evaluation software was used for the batch processing of measured data. The adjustment of the cantilever position above the sample was carried out under the microscope by controlling the position of the AFM-head by motorized stage equipped with Petri dish heater (JPK) allowing precise positioning of the sample together with a constant elevated temperature of the sample for the whole period of the experiment. Soft uncoated AFM probes HYDRA-2R-100N (Applied NanoStructures, Mountain View, CA, USA), i.e. silicon nitride cantilevers with silicon tips are used for stiffness studies because they are maximally gentle to living cells (not causing mechanical stimulation). Moreover, as compared with coated cantilevers, these probes are very stable under elevated temperatures in liquids – thus allowing long-time measurements without nonspecific changes in the measured signal.

### Wound healing assay

After the passage process, each cell line was re-suspended and seeded into a 24-well plate, the cell amount per well in 500 µl media being optimized to 150,000 for PC-3, 150,000 for PNT1A and 200,000 for 22Rv1. After 48 h, the cells were 100% confluent and scratched into the cell monolayer. After gentle wash and change of media, each well was photographed at time 0 and after 24 h on the same spot. The photos were analyzed according to instructions from the software creator^31^. The software computed the percentage of the open wound area. Each cell line was analyzed in min. twenty four repetitions.

### Real-time impedance-based cell proliferation and invasiveness assay

The impedance-based real-time cell analysis (RTCA) xCELLigence system was used according to supplier’s (Roche Applied Science and ACEA Biosciences, San Diego, CA, USA) instructions. The xCELLigence system consists of four main components: RTCA DP station, RTCA computer with integrated software and disposable CIM-plate 16. Firstly, seeding concentration optimal for proliferation and invasion assay was determined. Response optimal for the proliferation assay was found in the well containing 10,000 cells. After seeding a total number of cells in 200 μl of medium to each well in E-plate 16, attachment and proliferation of the cells were monitored every 15 min. For the invasiveness assay, optimal response was found in the well containing 20,000 cells. After coating the upper wells with Matrigel and after adding FBS as a chemoattractant, a total number of cells in 100 µl of medium to each well in CIM-plate 16 was seeded. Attachment and growth of the cells through the matrigel were monitored every 15 min. Duration of all experiments was 150 h. Relative invasiveness rate was defined as the cell index for matrigel-coated wells (cells need to decompose matrigel to produce signal) at a given time point. The impedance of electron flow caused by adherent cells is reported using a unitless parameter called Cell Index (CI), where CI = (impedance at time point n – impedance in the absence of cells)/nominal impedance value). In order to compare between cell lines/treatments, those cell indices were normalized to value 1.0 at the time when treatment was added.

### Colony forming assay

Colony forming assay is a method of choice to determine cell reproductive death after cytotoxic treatment. Only a fraction of seeded cells retains the capacity to produce colonies. Cells were harvested with trypsin in EDTA and centrifuged at 2700 rpm/7 min. Then the cells were re-suspended in the fresh medium and counted using CASY® Cell Counter. The cells were seeded onto 6–well plates. Each well contained 2 ml media and 100, 500, 1,000 or 2,000 cells. The plates were cultivated for 1–3 weeks. Optimal seeding was 500 cells. Any shaking or moving with plates was prevented to obtain clear colonies. The cells were subsequently fixed with cold methanol and visualised with the trypan blue.

### Quantitative phase imaging

Quantitative phase imaging was performed by using Tescan multimodal holographic microscope Q-PHASE. Cells were cultivated in Flow chambers μ-Slide I Lauer Family (Ibidi, Martinsried, Germany). To image a sufficient number of cells in one field of view, lens Nikon Plan 10/0.30 were chosen. Holograms were captured by CCD camera (XIMEA MR4021 MC-VELETA). Complete image reconstruction and image processing were performed in Q-PHASE control software. Cell dry mass values were derived according to^32,33^ from the phase, eq. (1)

1 $$m=\frac{\phi \lambda }{2\pi \alpha }$$

where m is cell dry mass density (in pg/μm^2^), φ is detected phase (in rad), λ is wavelength in μm (0.65 μm in Q-PHASE), and α is specific refraction increment (≈0.18 μm^3^/pg). All values in the formula except the Phi are constant. Phi (Phase) is the value measured directly by microscope.

### Statistical analysis and image processing

Quantitative phase images were analyzed with Q-PHASE control software, which includes segmentation based on watershed with region merging, followed by feature extraction (mass, circularity and position) for the following analysis. AFM and colony forming assay images were analyzed with MATLAB custom scripts. For the AFM images, segmentation masks were created by watershed segmentation of Setpoint Height images with manual corrections, then the masks were used for the extraction of mean cell values of both Setpoint Height and Young’s modulus images. For the colony forming assays, regions of interest were chosen by registration of each image to the reference image (with manually labeled area of interest). Next, the colonies were segmented by thresholding of the blue component of image transformed into Lab color space, where single fixed threshold was used. Finally, the fraction of areas covered by colonies was computed.

Fluorescence microscopy data were analyzed in ImageJ 1.52 h and Python 3.7.1 as follows: cells were manually segmented using actin fluorescence channel, two regions were created for analysis: whole cell and cell periphery, lining a 4 μm thick region around cell border and including most of periphery actin cytoskeleton. In these two regions following parameters were measured for both actin and tubulin fluorescence: Integrated intensity, median intensity, and following regions were measured to describe cell morphology: Cell area, Maximum caliper (max feret diameter), roundness, and aspect ratio. Moreover, stress fibers were manually segmented in every cell and following parameters were measured: number of fibers per cell, feret angle of fiber, integrated intensity, fiber length, mean intensity. Next, a standard deviation of feret angles of individual fibers was calculated relatively to mean of feret angle using a circstd function from scipy package for Python.

Data were checked for normality, based on which either paired ANOVA or Kruskal–Wallis test were applied in order to test the impact of the tested factors (cell line, resistance, treatment), and either Pearson or Spearman correlation were applied in order to test dependency between variables. MATLAB 2017a (Statistics and Machine Learning Toolbox) was used for this statistical analysis with p < 0.05 considered as significant.

## Results

### Biomechanical profiling of non-treated prostate cells

**Figure 1:**
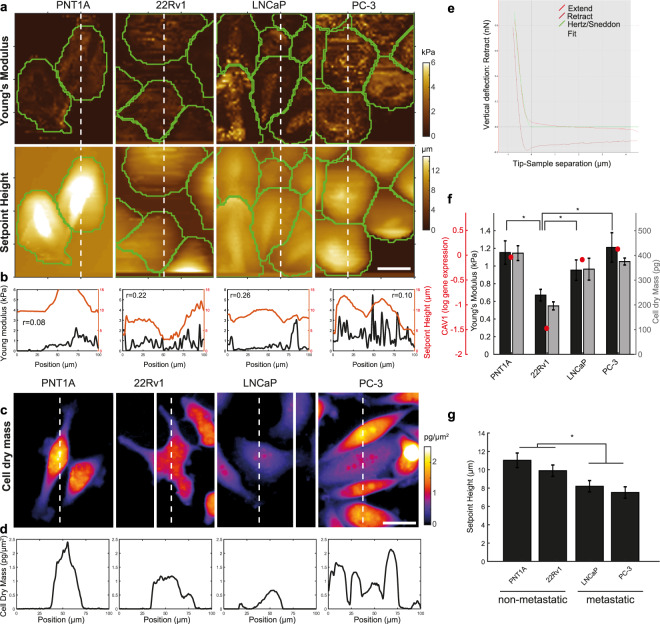
Cell stiffness, cell dry mass and CAV1 expression of untreated prostate cancer cell lines. (**a**) Cell stiffness maps determined by indentation (Young’s modulus) of prostatic cells (first row) and cell height (displayed as Setpoint Height, second row). (**b**) Profile of Setpoint Height/Young’s modulus of red/blue lines shown in A together with pixel Pearson correlation values. (**c**) Cell dry mass. (**d**) Profile of cell dry mass in the corresponding cutting point (white line). (**e**) Hertz model fitting to a force curve obtained on PC-3 cells. (**f**) Values of Young’s modulus, Cell dry mass, CAV1 gene expression for prostatic cells. Statistical significance shown for Young’s modulus only. (**g**) Setpoint Height of cells. Significance between metastatic and non-metastatic cells highlighted. Calibration bars for A and C represent 25 μm. Error bars denote standard errors. Asterisk indicates statistical significance at p < 0.05. For detailed statistics see Supplementary Table S1.

Force-indentation curves were successfully acquired for 68 LNCaP, 42 PC-3, 20 22Rv1 and 64 PNT1A non-treated cells to which the Hertz model was fitted. The Hertz model worked well in the used indentation range (example in Fig. 1e for PC-3 cells). Coherence-controlled holograms were successfully acquired for 104 LNCaP, 17 PC-3, 99 22Rv1 and 77 PNT1A cells. Figure 1a,b (AFM) and Fig. 1c,d (coherence-controlled holographic microscopy) show representative profiles of each of the cell lines used in this work. The median Young’s moduli values, cell mass values and *CAV1* gene expression obtained for these four cell lines are shown in Fig. 1f. The values of Young’s moduli were within the range reported in the literature^9^. The median values of Young’s moduli were E = 997 Pa for LNCaP, 1210 Pa for PC-3, 1153 Pa for PNT1A and 671 Pa for 22Rv1. The Young’s moduli obtained for the 22Rv1 cells were significantly lower than those obtained for the PC-3, PNT1A and LNCaP cells (p ≤ 0.009 in all cases, for details, see Supplementary Tab. S1). On the other hand, no significant changes in cell stiffness were found between the PC-3, PNT1A and LNCaP cells. The observed values of Young’s moduli followed similar trends with cell dry mass measured by coherence-controlled holographic microscopy and with *CAV1* gene expression (see Fig. 1f).

Significant cell height (Setpoint Height) differences were observed between the PNT1A and PC-3 cells (p ≤ 0.001), PNT1A and LNCaP cells (p = 0.001) and between 22Rv1 and PC-3 cells (p = 0.039). Cells of metastatic origin were significantly flatter than cells derived from a primary tumour or benign tissue (p ≤ 0.001, Fig. 1g). The LNCaP cells also showed correlation between cell height and cell stiffness stronger than the other types of cells (Fig. 1b).

### Effect of cytostatics on cell biomechanics and morphology

**Figure 2:**
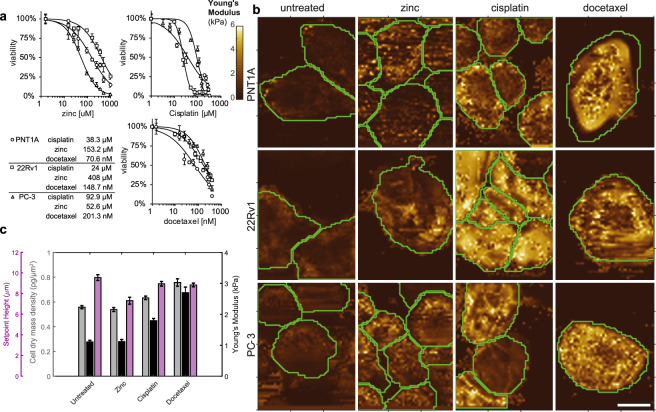
Changes in biomechanical features; effect of treatment. (**a**) MTT test and respective IC50 concentration values. For detailed statistics see Supplementary Table S1. (**b**) Representative Young’s moduli maps of cell lines treated with zinc(II), docetaxel, and cisplatin. (**c**) Young’s moduli (cell stiffness), Setpoint Height, and cell dry mass density changes after zinc(II), docetaxel and cisplatin treatment. All tested cell lines (PNT1A, 22Rv1 and PC-3) assessed together. Error bars denote standard errors.

We analyzed cell stiffness, cell dry mass, and cell height in cells that had survived the chosen 24 h treatments (1x IC50 of zinc(II), cisplatin, and docetaxel; for MTT see Fig. 2a). Force-indentation curves were successfully acquired for 27 PC-3, 41 22Rv1 and 84 PNT1A cisplatin-treated cells, for 23 PC-3, 23 22Rv1 and 23 PNT1A zinc(II) treated cells, and for 83 PC-3, 26 22Rv1 and 61 PNT1A docetaxel-treated cells. Figure 2b shows representative profiles for each treatment. Coherence-controlled holograms were successfully acquired for 118 PC-3, 187 22Rv1 and 33 PNT1A cisplatin-treated cells, for 61 PC-3, 67 22Rv1 and 37 PNT1A zinc(II) treated cells, and for 19 PC-3, 107 22Rv1 and 39 PNT1A docetaxel-treated cells.

**Figure 3:**
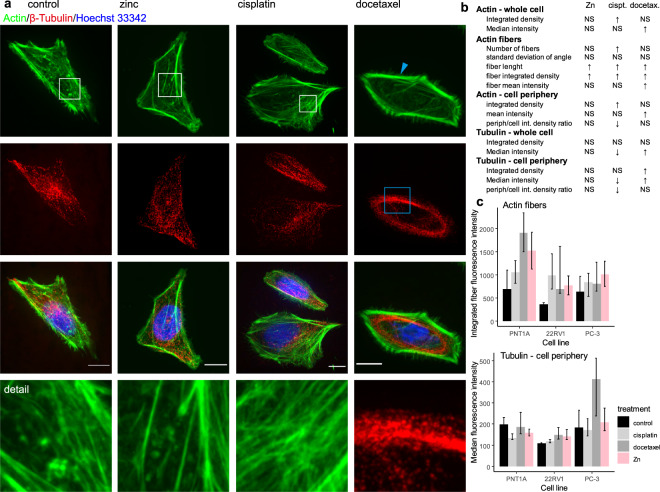
β-Tubulin; effect of treatment, PNT1A cells. (**a**) Tubulin fibers form a thick cables and dense shell due to docetaxel treatment (see blue square). In contrast to docetaxel that caused significant changes in microtubules, cisplatin affected actin cytoskeleton more fundamentally. Accumulation of stress fibers and/or changes in actin organization were observed. All three treatments (zinc, cisplatin, and docetaxel) caused stress fibers accumulation and/or actin disintegration in some cells (see and compare areas in the white squares), but cisplatin was the most effective in this process (all images (N = 242) and data analyses are accessible on (https://zenodo.org/, Digital Object Identifiers: 10.5281/zenodo.1494935). For remaining cell lines see Supplementary Fig. S1. Cells are shown at 100x magnification, calibration bar indicates 10 μm, detail square width 10 μm. (**b**) Results of image analysis for actin and tubulin structures, shown results for all cell lines, for detailed results of all cell lines see Supplementary Table S2 (**c**) Actin fibers, Tubulin in cell periphery (defined as a region 4 μm from the cell border). Intensity of actin fibers localized in cytoplasm (i.e. those fibers not in periphery) is increased by cisplatin treatment, tubulin fluorescence is pronounced in cell periphery after docetaxel treatment. Displayed as median and interquartile range.

Because no significant changes in cell stiffness, cell flatness, and cell dry mass were found between the metastatic cell lines PC-3 and LNCaP, only the more aggressive PC-3 cell line was included in further experiments. In all tested cell lines (PNT1A, 22Rv1 and PC-3), cells treated with the cytostatic drugs (cisplatin or docetaxel) had a higher Young’s modulus (were stiffer) as compared to non-treated cells (p ≤ 0.007 in all tested cell lines). Docetaxel increased cell stiffness more effectively than cisplatin (p ≤ 0.001). On the other hand, this effect was not observed in the zinc(II) treated cells (see Fig. 2b,c and Supplementary Tab. S1). Consequently, changes in cell stiffness due to cisplatin treatment do not result from simple metal accumulation in the cells (platinum or zinc) but are connected with changes in the cytoskeletal organization (for illustration see Fig. 3 and Supplementary Fig. S1 with fluorescent staining of tubulin and actin). Using an image analysis, it was observed, that cisplatin causes significant increase of actin density (determined by increased integrated density of actin fluorescence) and in particular, increased number of stress fibers, their increased length and fluorescent intensity (Fig. 3b,c and Supplementary Table S2). With regard to tubulin, no increase of cell periphery tubulin content was observed after cisplatin treatment. On the other hand, docetaxel treatment caused distinct accumulation of tubulin in cell periphery (described by increased median and integrated fluorescence density). Elongation of actin fibers was observed as a non-specific marker of cell stress, caused by all types of treatments used in this study.

Cell dry mass density values measured by coherence-controlled holographic microscopy followed the trend of Young’s modulus. On the other hand, no significant changes were observed in cell height due to the cytostatic treatment (values of Setpoint Height) (Fig. 2c).

**Figure 4:**
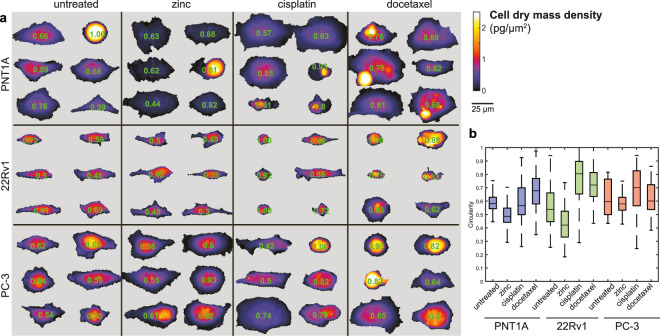
Cell dry mass distribution and morphology; effect of treatment. (**a**) Matrix of representative cells, coherence-controlled holographic microscopy. Longer axis orientation of the cells was unified. Numbers inside cells represent circularity, 4π(area/perimeter^2^). (**b**) Circularity of the cells. Boxes and error bars represent interquartile range and 95% percentile.

Next, the association was analyzed between morphological parameters, cell treatment and the cell type (Fig. 4). Cell circularity differed distinctly and tended to be significantly lower due to the zinc treatment and higher due to the cisplatin and docetaxel treatments in the 22Rv1 and PNT1A cells. However, no significant association between the treatments and cell circularity was observed in the metastatic PC-3 cells.

### Migration, invasiveness and cytostatic treatment

**Figure 5:**
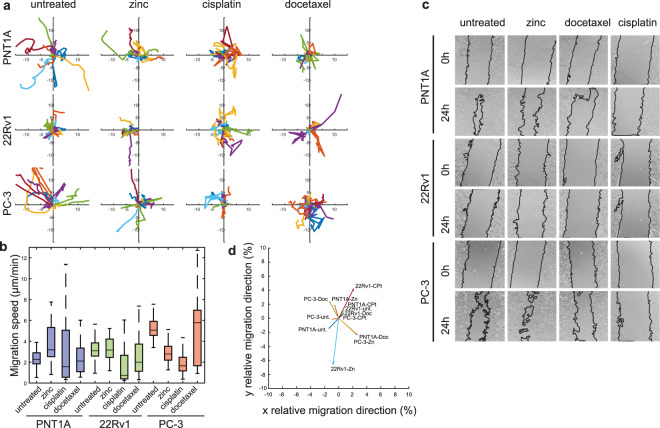
Changes in motility of prostate cancer cells; effect of treatment. (**a**) Rose diagram of cell migration speed obtained by coherence-controlled holographic microscopy and corresponding bar charts, (**b**) Boxes and error bars represent interquartile range and 95% percentile. (**c**) Wound-healing assay in t = 0 and 24 h. (**d**) Summary vector of the movements of all cells after respective treatments divided by migrated path length.

To establish the relationship between the changes in cell stiffness and the metastatic potential of prostate cancer cells, we performed invasion assay, colony forming assay and wound healing assay of cells that had survived the cisplatin, docetaxel and zinc treatment, and of non-treated cells. Simple migration ability was tested by wound healing assay and verified by real-time coherence-controlled holographic microscopy (Fig. 5). Due to the cisplatin and docetaxel treatment, a significant reduction of cell migration was observed in all tested cell lines according to the wound-healing assay. This trend was observable also in the coherence-controlled migration assay, but did not gain statistical significance for PNT1A and PC-3. On the other hand, the zinc treatment enhanced the migration speed in the PNT1A cells (Fig. 5a,b). The circularity of the cells was in the negative correlation with the migration speed after the zinc and cytostatic treatments (r_Sp_ = −0.33; p = 0.0001 for zinc and r_Sp_ = −0.48; p = 0.0001 for cytostatic drugs). On the other hand, the cell circularity showed no association with the cell migration speed in the non-treated cells.

**Figure 6:**
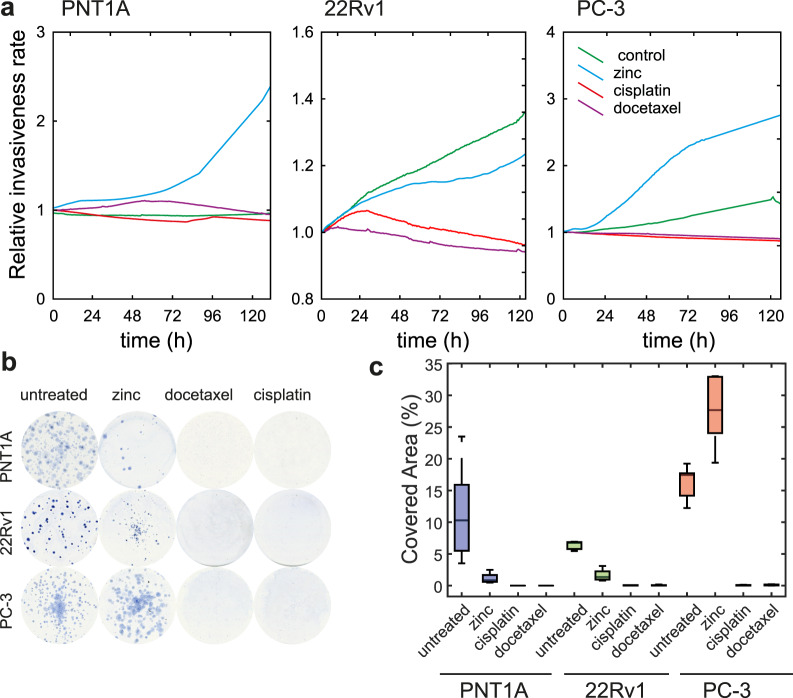
Changes in the invasive potential of prostate cancer cells; effect of treatment. (**a**) Impedance-based real-time cell analysis (xCELLigence invasion assay); effect of zinc(II), docetaxel, and cisplatin treatment. (**b**) Representative images of colony forming assays and calculated covered area, (**c**) Boxes and error bars represent interquartile range and 95% percentile.

Furthermore, the ability of cancer cells to spread to the surrounding tissues was tested by using the label-free impedance-based real-time cell analysis, where the cells must be able to go through the matrigel. Due to the cisplatin and docetaxel treatment, a significant reduction of cell invasion was observed (Fig. 6a). Conversely, the long-term zinc treatment enhanced the ability to invade, for the PC-3 cells in particular. To determine the reproductive death of cells that had survived the cytotoxic treatment, the colony forming assay was used. A significant reduction in colony formation was observed due to the cisplatin and docetaxel treatment, where no formation of new colonies was observed. Conversely, the long-term zinc treatment enhanced the ability to form colonies in the metastatic PC-3 cells (Fig. 6b,c).

## Discussion

Tumour transformed cells differ from normal tissue-connected cells in many features. Significant observable differences were shown in biomechanical properties such as cell adhesion and mechanical stiffness^12,34–36^. Accordingly, cell stiffness studies showed that cancerous cells are usually less stiff than their normal counterparts^37–39^. This observation was only partially confirmed in our study. Whereas tumour cells derived from the primary tumour tissue (22Rv1 cells) were significantly softer than cells derived from the normal prostatic tissue (PNT1A cells), this was not true for metastatic cell lines (PC-3 and LNCaP).

In many studies, it was demonstrated that the low cell stiffness could serve as a marker for cell motility and malignant potential^9–11^. Nevertheless, in our case, both types of cells derived from metastatic sites (PC-3-high invasiveness, LNCaP-low invasiveness) were stiffer and flatter than tumour cells derived from the primary tumour tissue. Our results also suggest some role of CAV1 in the total stiffness of prostate cancer cells and a positive correlation between cell stiffness and cell dry mass in the non-treated cells and between cell stiffness and cell dry mass density in the treated cells. The influence of CAV1 could be managed by modulating the Rho/ROCK pathways^22^. Other studies focused on the prostate-derived cells had similar results regarding the cell stiffness^11,34,40,41^.

According to our results, cytoskeleton plays a key role in the changes of biomechanical features of cancer cells, because the treatment with docetaxel that stabilizes microtubule and blocks their dynamics caused a significant enhancement of the cell stiffness. This observation is in a good agreement with other studies^4,14,15^ and interestingly, the cytochalasin that is known to depolymerize the actin filaments, caused a decrease of the cell stiffness^12,42^. Furthermore, the treatment with cisplatin caused also a significant increase in the cell stiffness of prostate cancer cells and the effect of cisplatin on cytoskeleton as mentioned in^20^ and^21^ was confirmed. Changes in cell stiffness due to cisplatin treatment probably do not result from simple metal accumulation in the cells because no such increase was shown in the zinc-treated cells. The observed reorganization of the cell cytoskeleton implies changes in cancer cell motility and invasiveness. Accordingly, a significant decrease in cell migration, invasion and forming of colonies was observed in cells that had survived the docetaxel and cisplatin treatments in all tested cell lines. We can speculate that this decrease is among other things a consequence of increasing cell stiffness because highly invasive cells need to be rather more pliable^9,13,37^. Consequently, changes in cell stiffness could be an important overlooked effect of antineoplastic drugs. Conversely, the zinc(II) treatment did not show such clear trends as the treatments with cisplatin and docetaxel. The effect of zinc treatment was highly influenced by the type of cell line.

## Conclusions

In this study, we demonstrated AFM together with coherence-controlled holographic microscopy to bring a promising approach that helps understand the correlation between the cell structure, cell mechanics, and function (changes in migration speed, cell dry mass, cell circularity, etc.). Despite the differences in the absolute value of Young’s modulus across biomechanical studies, the obtained relative changes of Young’s modulus were shown to be consistent.

Both cisplatin and docetaxel treatments caused a significant increase in the cell stiffness of prostate cancer cells that had survived the treatment. Hence, we presume the effect of cisplatin on the cytoskeleton. Consequently, the decrease in cell migration, invasion and forming of colonies observed in cells surviving the docetaxel and cisplatin treatment was associated with the increasing cell stiffness. We maintain therefore that changes in the cell stiffness could be an important overlooked effect of cisplatin-based anti-cancer drugs.

## Supplementary information

Supplementary Table S1, Table S2, Supplemntary Figure S1

## Data Availability

Atomic force microscopy and confocal microscopy data images used in our study are available for download in the Zenodo repository (https://zenodo.org/, Digital Object Identifiers: 10.5281/zenodo.1494935).
