# Author Correction: Cisplatin enhances cell stiffness and decreases invasiveness rate in prostate cancer cells by actin accumulation

**DOI:** 10.1038/s41598-022-23540-y

**Published:** 2022-11-04

**Authors:** Martina Raudenska, Monika Kratochvilova, Tomas Vicar, Jaromir Gumulec, Jan Balvan, Hana Polanska, Jan Pribyl, Michal Masarik

**Affiliations:** 1 Department of Physiology, Faculty of Medicine, Masaryk University, Kamenice 5, 625 00 Brno, Czech Republic; 2 Department of Pathological Physiology, Faculty of Medicine, Masaryk University, Kamenice 5, 625 00 Brno, Czech Republic; 3 Department of Biomedical Engineering, Faculty of Electrical Engineering and Communication, Brno University of Technology, Technicka 3058/10, 616 00 Brno, Czech Republic; 4 Central European Institute of Technology, Brno University of Technology, Technicka 3058/10, 616 00 Brno, Czech Republic; 5 Central European Institute of Technology, Masaryk University, Kamenice 5, 625 00 Brno, Czech Republic

Correction to: *Scientific Reports* 10.1038/s41598-018-38199-7, published online 07 February 2019

This Article contains an error in Figure 5c, where the image “22Rv1 - cisplatin 24 h” is a duplication of the image “22Rv1 - cisplatin 0 h”. The correct Figure 5 and the accompanying legend appear below.

**Figure 5:**
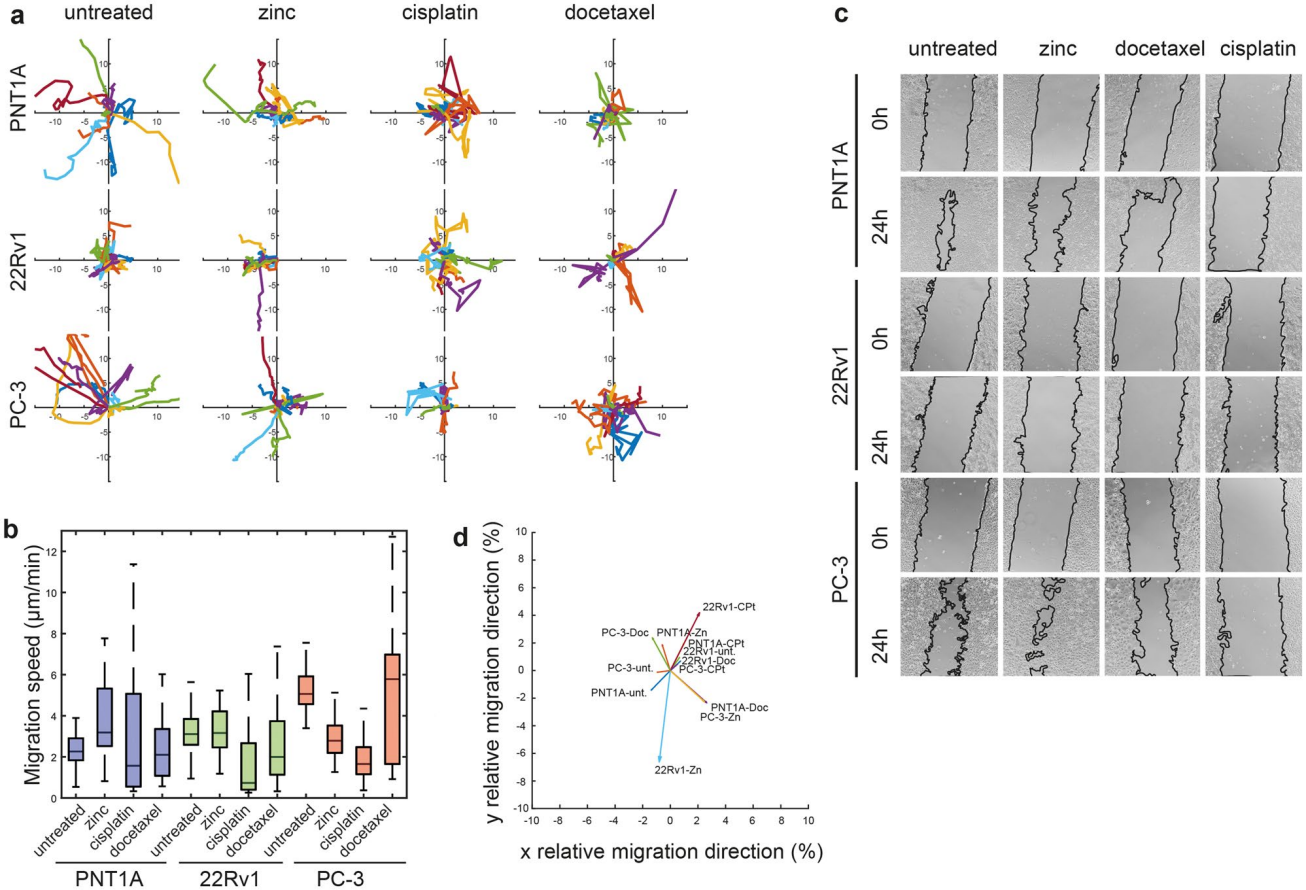
Changes in motility of prostate cancer cells; effect of treatment. (**a**) Rose diagram of cell migration speed obtained by coherence-controlled holographic microscopy and corresponding bar charts, (**b**) Boxes and error bars represent interquartile range and 95% percentile. (**c**) Wound-healing assay in t = 0 and 24 h. (**d**) Summary vector of the movements of all cells after respective treatments divided by migrated path length.

