# Supplementary material for: Cisplatin enhances cell stiffness and decreases invasiveness rate in prostate cancer cells by actin accumulation
Source: Sci Rep. 2019 Feb 7;9:1660. doi: 10.1038/s41598-018-38199-7 (PMC6367361; doi:10.1038/s41598-018-38199-7)
Supplement: Supplementary file 1 — Supplementary Table S1, Table S2, Supplemntary Figure S1 [file 41598_2018_38199_MOESM1_ESM.pdf]

Cisplatin enhances cell stiffness and decreases invasiveness rate in prostate cancer cells by actin accumulation

Martina Raudenska[1×], Monika Kratochvilova[1×], Tomas Vicar[2,3], Jaromir Gumulec[1,2,4], Jan Balvan[1,2,4], Hana Polanska[2], Jan Pribyl[5], and Michal Masarik[1,2,4]*

[1]Department of Physiology, Faculty of Medicine, Masaryk University / Kamenice 5, CZ-625 00 Brno, Czech Republic

[2]Department of Pathological Physiology, Faculty of Medicine, Masaryk University / Kamenice 5, CZ-625 00 Brno, Czech Republic

[3]Department of Biomedical Engineering, Faculty of Electrical Engineering and Communication, Brno University of Technology, Technicka 3058/10, CZ-616 00 Brno, Czech Republic

[4]Central European Institute of Technology, Brno University of Technology, Technicka 3058/10, CZ-616 00 Brno, Czech Republic

[5]Central European Institute of Technology, Masaryk University/ Kamenice 5, CZ-625 00 Brno, Czech Republic

*Dr. Michal Masarik, Department of Physiology and Pathological Physiology, Faculty of Medicine, Masaryk University, Kamenice 5, CZ-625 00 Brno, Czech Republic; E-mail: masarik@med.muni.cz Phone: +420-5-4949-3631; fax: +420-5-4949-4340

[×]Martina Raudenska and Monika Kratochvilova contributed equally to this work.

Supplementa

Table S1: Summary of statistics used throughout the study
(page 2)
Figure S1: Confocal microscopy of cell lines exposed to cisplatin, docetaxel and zinc.
(page 5)
Table S2: Changes of actin and tubulin, analysis of confocal microscopy
(page 7)

Table S1: Summary of statistics used throughout the study. Values shown as median and interquartile range (IQR). Lilliefors test used for normality analysis. * indicates Kruskal-Wallis test used instead of ANOVA for this comparison. † for colony forming analysis, N reflects number of chamber wells instead of the number of analyzed cells as in the other analyses.

Untreated cells

				normality	ANOVA/KW p value		
**Modality**	**Cell line**	**median (IQR)**	**N**	**p**	**vs. 22Rv1**	**vs. LNCaP**	**vs. PC-3**
Young Modulus (kPa)							
	PNT1A	1.15 (0.64-1.53)	64	0.221	0.001	0.763	0.343
	22Rv1	0.67 (0.39-0.81)	20	0.500		0.009	<0.001
	LNCaP	0.95 (0.68-1.36)	68	0.003			0.053
	PC-3	1.21 (0.78-1.74)	42	0.374			
Cell Mass (pg/μm^2)*							
	PNT1A	409.34 (358.62-517.73)	77	0.001	<0.001	0.019	0.889
	22Rv1	196.06 (171.27-223.95)	99	0.001		<0.001	<0.001
	LNCaP	344.46 (281.12-489.13)	104	0.001			0.786
	PC-3	375.34 (362.25-419.63)	17	0.337			
Setpoint height (µm)*							
	PNT1A	11.03 (8.73-13.26)	64	0.001	0.698	0.001	<0.001
	22Rv1	9.89 (7.48-11.61)	20	0.026		0.437	0.039
	LNCaP	8.2 (6.88-11.05)	68	0.104			0.304
	PC-3	7.52 (5.8-9.54)	42	0.237			

Treated cells cells

					normality	ANOVA/KW p value		
**Modality**	**treatment**	**Cell line**	**median (IQR)**	**N**	**p**	**vs. zinc**	**vs. cisplatin**	**vs. docetaxel**
Circularity (0-1)								
	untreated	PNT1A	0.58 (0.54-0.63)	77	0.285	0.002	0.701	<0.001
	zinc	PNT1A	0.49 (0.44-0.55)	37	0.205		<0.001	<0.001
	cisplatin	PNT1A	0.57 (0.50-0.70)	33	0.372			0.025
	docetaxel	PNT1A	0.68 (0.58-0.77)	39	0.500			
	untreated	22Rv1	0.54 (0.45-0.66)	99	0.055	<0.001	<0.001	<0.001
	zinc	22Rv1	0.42 (0.33-0.52)	67	0.327		<0.001	<0.001
	cisplatin	22Rv1	0.80 (0.65-0.90)	187	0.001			0.752
	docetaxel	22Rv1	0.72 (0.64-0.81)	107	0.500			
	untreated	PC-3	0.60 (0.50-0.76)	17	0.500	0.608	0.305	1.000
	zinc	PC-3	0.58 (0.53-0.63)	61	0.500		<0.001	0.599
	cisplatin	PC-3	0.70 (0.57-0.83)	118	0.249			0.243
	docetaxel	PC-3	0.60 (0.54-0.72)	19	0.412			
Migration speed (µm/min)*								
	untreated	PNT1A	2.26 (1.84-2.90)	77	0.001	0.011	0.768	0.779
	zinc	PNT1A	3.19 (2.53-5.33)	37	0.001		0.003	0.003
	cisplatin	PNT1A	1.57 (0.55-5.07)	33	0.001			1.000
	docetaxel	PNT1A	2.1 (1.08-3.35)	39	0.005			
	untreated	22Rv1	3.11 (2.59-3.84)	99	0.001	0.961	<0.001	0.006
	zinc	22Rv1	3.16 (2.46-4.21)	67	0.001		<0.001	0.003
	cisplatin	22Rv1	0.73 (0.40-2.67)	187	0.001			<0.001
	docetaxel	22Rv1	2.00 (1.13-3.74)	107	0.001			

untreated	PC-3	5.06 (4.56-5.91)	17	0.011	0.005	<0.001	0.377
zinc	PC-3	2.79 (2.19-3.52)	61	0.001		<0.001	0.467
cisplatin	PC-3	1.66 (1.16-2.47)	118	0.001			<0.001
docetaxel	PC-3	5.78 (1.65-6.98)	19	0.379			
Colony-forming assay (% covered area)*†							
untreated	PNT1A	10.29 (5.50-15.90)	6	N/A	0.410	<0.001	0.001
zinc	PNT1A	0.85 (0.60-1.67)	6	N/A		0.069	0.126
cisplatin	PNT1A	0	6	N/A			0.994
docetaxel	PNT1A	0	6	N/A			
untreated	22Rv1	6.73 (5.78-6.87)	3	N/A	0.734	0.018	0.007
zinc	22Rv1	1.36 (0.96-2.26)	6	N/A		0.091	0.035
cisplatin	22Rv1	0.04 (0.01-0.09)	6	N/A			0.982
docetaxel	22Rv1	0.02 (0.00-0.08)	6	N/A			
untreated	PC-3	17.44 (14.19-17.71)	6	N/A	0.336	0.108	0.472
zinc	PC-3	27.68 (24.04-32.91)	6	N/A		<0.001	0.025
cisplatin	PC-3	0.04 (0.00-0.10)	6	N/A			0.978
docetaxel	PC-3	0.10 (0.03-0.19)	3	N/A			
Cell Mass (pg/μm^2)*							
Untreated	PNT1A	409.34 (358.62-517.73)	77	0.001	0.663	0.002	0.001
Zinc	PNT1A	386.18 (270.74-542.99)	37	0.382		0.125	0.111
Cisplatin	PNT1A	251.47 (180.42-445.67)	33	0.015			1.000
Docetaxel	PNT1A	292.58 (195.00-449.81)	39	0.37			
Untreated	22Rv1	196.06 (171.27-223.95)	99	0.001	<0.001	<0.001	0.435
Zinc	22Rv1	271.41 (211.34-548.02)	67	0.001		<0.001	<0.001
Cisplatin	22Rv1	100.35 (73.54-170.18)	187	0.001			<0.001
Docetaxel	22Rv1	179.13 (111.92-272.18)	107	0.001			
Untreated	PC-3	375.34 (362.25-419.63)	17	0.337	<0.001	<0.001	<0.001
Zinc	PC-3	492.55 (396.37-553.06)	61	0.001		0.997	0.656
Cisplatin	PC-3	416.27 (335.15-540.55)	118	0.004			0.853
Docetaxel	PC-3	569.54 (485.98-757.39)	19	0.182			
Untreated	together	304.14 (192.15-408.32)	193	0.001	<0.001	<0.001	0.014
Zinc	together	423.19 (266.39-553.06)	165	0.001		<0.001	<0.001
Cisplatin	together	226.93 (91.44-407.98)	338	0.001			0.951
Docetaxel	together	226.60 (130.55-354.48)	165	0.001			
Young Modulus (kPa)*							
Untreated	PNT1A	1.15 (0.64-1.53)	64	0.221	0.536	<0.001	<0.001
Zinc	PNT1A	0.85 (0.61-1.23)	23	0.150		<0.001	<0.001
Cisplatin	PNT1A	1.68 (1.24-2.09)	84	0.500			0.011
Docetaxel	PNT1A	2.72 (1.60-3.78)	61	0.001			
Untreated	22Rv1	0.67 (0.39-0.81)	20	0.500	0.275	<0.001	<0.001
Zinc	22Rv1	0.97 (0.71-1.21)	23	0.257		0.014	<0.001
Cisplatin	22Rv1	1.97 (1.01-3.00)	41	0.015			0.157
Docetaxel	22Rv1	2.42 (1.69-5.67)	26	0.001			
Untreated	PC-3	1.21 (0.78-1.74)	42	0.374	0.433	0.007	<0.001
Zinc	PC-3	1.65 (1.35-2.06)	23	0.500		0.483	0.009
Cisplatin	PC-3	1.98 (1.72-2.75)	27	0.500			0.432
Docetaxel	PC-3	2.88 (1.42-4.63)	83	0.009			
Untreated	together	1.11 (0.62-1.46)	126	0.030	0.961	<0.001	<0.001
Zinc	together	1.12 (0.71-1.52)	69	0.127		<0.001	<0.001

	Cisplatin	together	1.80 (1.21-2.43)	152	0.123			<0.001
	Docetaxel	together	2.70 (1.55-4.65)	170	0.001			
Setpoint height (μm)*								
	Untreated	PNT1A	11.03 (8.73-13.26)	64	0.001	<0.001	0.048	0.040
	Zinc	PNT1A	6.67 (3.71-9.37)	23	0.033		0.001	0.003
	Cisplatin	PNT1A	9.57 (7.95-11.28)	84	0.022			0.993
	Docetaxel	PNT1A	9.32 (8.33-10.89)	61	0.486			
	Untreated	22Rv1	9.89 (7.48-11.61)	20	0.026	0.709	0.799	0.087
	Zinc	22Rv1	8.95 (6.98-10.24)	23	0.134		0.991	0.563
	Cisplatin	22Rv1	9.54 (5.96-12.01)	41	0.053			0.275
	Docetaxel	22Rv1	8.54 (5.47-9.92)	26	0.083			
	Untreated	PC-3	7.52 (5.80-9.54)	42	0.237	0.784	0.895	0.503
	Zinc	PC-3	6.38 (5.24-9.20)	23	0.019		0.995	0.136
	Cisplatin	PC-3	8.29 (5.57-8.91)	27	0.002			0.196
	Docetaxel	PC-3	8.43 (6.44-10.13)	83	0.377			
	Untreated	all	9.56 (7.33-12.39)	126	0.018	<0.001	0.680	0.092
	Zinc	all	7.32 (5.49-9.65)	69	0.062		0.002	0.038
	Cisplatin	all	8.96 (7.58-11.16)	152	0.132			0.597
	Docetaxel	all	8.85 (6.79-10.42)	170	0.018			

KW, Kruskall-Wallis test, Lilliefors test used for normality analysis. * indicate Kruskal-Wallis test used instead of ANOVA for this comparison. † for colony-forming analysis, N reflect number of chamber wells instead of number of analyzed cells as in other analyses.

Figure S1: Confocal microscopy of cell lines exposed to cisplatin, docetaxel and zinc. Max intensity projections of representative cells. Scalebar represents 20 µm.

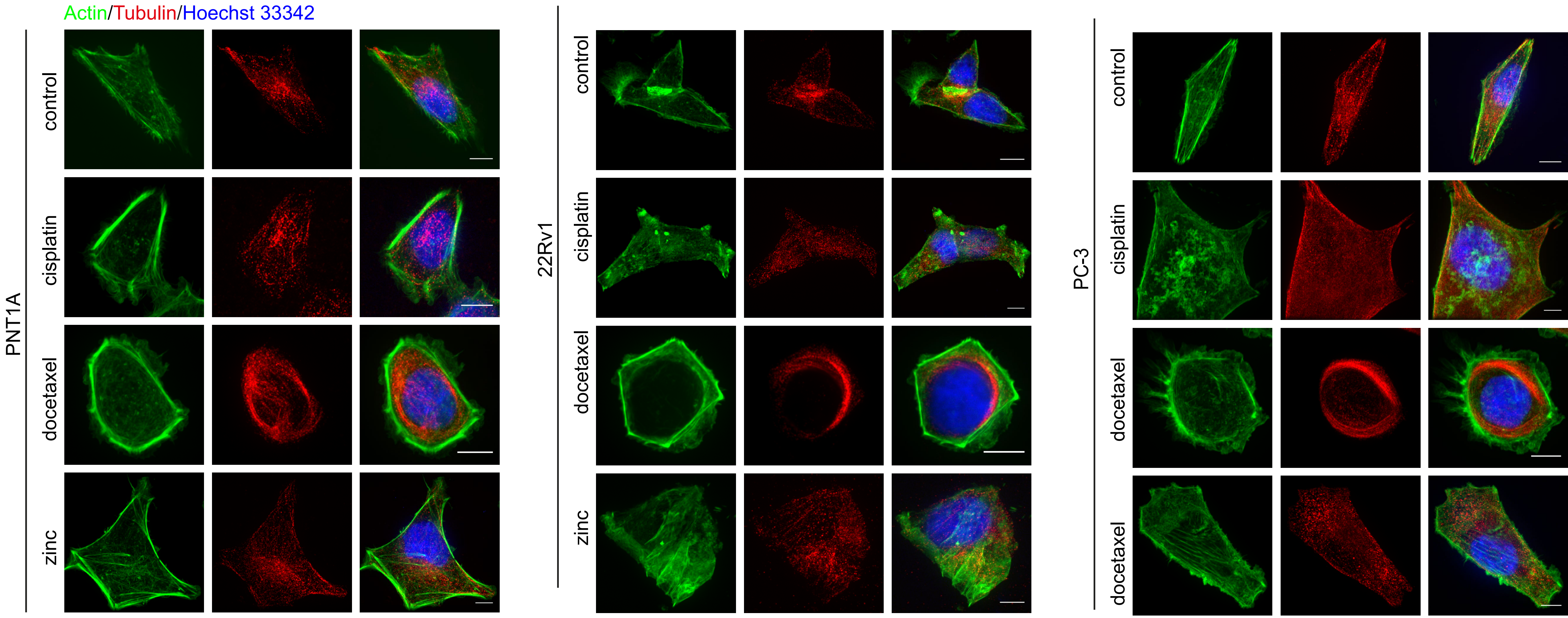

Figure S1: Confocal microscopy of cell lines exposed to cisplatin, docetaxel and zinc. Max intensity projections of representative cells. Scalebar represents 20 μm.

Table S2: Changes of actin and tubulin, analysis of confocal microscopy. Results of linear model of actin and tubulin staining for following cellular regions: whole cell, cell periphery (defined by a range 4 μm from the cell border), and fibers. SD of angle, standard deviation of actin fiber angles standardized to the mean angle.For description of measured factors, see Methods section of article. "Estimate" shown as relative change compared to intercept – control, untreated cells.

Cell line	structure	substructure	factor	level	Estimate	Std. Error	Pr(>|t|)
**all**	actin	cell	Integrated density	control	894145.0	121694.0	0.000
**all**	actin	cell	Integrated density	cisplatin	417669.0	156832.0	0.008
**all**	actin	cell	Integrated density	docetaxel	109809.0	149208.0	0.462
**all**	actin	cell	Integrated density	Zn	169941.0	156832.0	0.279
**all**	actin	cell	Median intensity	control	289.1	14.5	< 2e-16
**all**	actin	cell	Median intensity	cisplatin	4.0	18.7	0.829
**all**	actin	cell	Median intensity	docetaxel	50.4	17.8	0.005
**all**	actin	cell	Median intensity	Zn	-1.6	18.7	0.932
**all**	actin	fibers	Number of fibers	control	2.0	0.3	0.000
**all**	actin	fibers	Number of fibers	cisplatin	2.0	0.4	0.000
**all**	actin	fibers	Number of fibers	docetaxel	-0.4	0.4	0.240
**all**	actin	fibers	Number of fibers	Zn	0.4	0.4	0.358
**all**	actin	fibers	SD of angle	control	21.3	2.7	0.000
**all**	actin	fibers	SD of angle	cisplatin	2.9	3.1	0.365
**all**	actin	fibers	SD of angle	docetaxel	0.3	3.2	0.917
**all**	actin	fibers	SD of angle	Zn	-0.9	3.2	0.776
**all**	actin	fibers	Fiber lenght (μm)	control	4.7	0.5	< 2e-16
**all**	actin	fibers	Fiber lenght (μm)	cisplatin	1.9	0.6	0.002
**all**	actin	fibers	Fiber lenght (μm)	docetaxel	3.3	0.6	0.000
**all**	actin	fibers	Fiber lenght (μm)	Zn	3.5	0.6	0.000
**all**	actin	fibers	Integrated density	control	732.1	130.5	0.000
**all**	actin	fibers	Integrated density	cisplatin	358.5	159.8	0.026
**all**	actin	fibers	Integrated density	docetaxel	919.9	159.5	0.000
**all**	actin	fibers	Integrated density	Zn	635.2	161.5	0.000
**all**	actin	fibers	Mean intensity	control	400.4	29.5	< 2e-16
**all**	actin	fibers	Mean intensity	cisplatin	25.4	36.1	0.482
**all**	actin	fibers	Mean intensity	docetaxel	121.1	36.0	0.001
**all**	actin	fibers	Mean intensity	Zn	21.0	36.5	0.565
**all**	actin	periphery	Integrated density	control	596532.0	51021.0	< 2e-16
**all**	actin	periphery	Integrated density	cisplatin	228582.0	65753.0	0.001
**all**	actin	periphery	Integrated density	docetaxel	100667.0	62557.0	0.108
**all**	actin	periphery	Integrated density	Zn	92588.0	65753.0	0.160
**all**	actin	periphery	Median intensity	control	305.2	16.6	< 2e-16
**all**	actin	periphery	Median intensity	cisplatin	11.4	21.3	0.593
**all**	actin	periphery	Median intensity	docetaxel	69.2	20.3	0.001
**all**	actin	periphery	Median intensity	Zn	5.5	21.3	0.796
**all**	actin	periphery	Periphery/cell integrated density ratio	control	0.8	0.0	< 2e-16
**all**	actin	periphery	Periphery/cell integrated density ratio	cisplatin	-0.1	0.0	0.002
**all**	actin	periphery	Periphery/cell integrated density ratio	docetaxel	0.0	0.0	0.192
**all**	actin	periphery	Periphery/cell integrated density ratio	Zn	0.0	0.0	0.100
**all**	morphology	cell	Aspect ratio	control	2.1	0.1	<2e-16
**all**	morphology	cell	Aspect ratio	cisplatin	0.2	0.1	0.275

**all**	morphology	cell	Aspect ratio	docetaxel	-0.2	0.1	0.170
**all**	morphology	cell	Aspect ratio	Zn	0.1	0.1	0.695
**all**	morphology	cell	Cell area (μm^2)	control	462.4	54.4	0.000
**all**	morphology	cell	Cell area (μm^2)	cisplatin	189.8	70.1	0.007
**all**	morphology	cell	Cell area (μm^2)	docetaxel	-35.1	66.7	0.599
**all**	morphology	cell	Cell area (μm^2)	Zn	48.6	70.1	0.489
**all**	morphology	cell	Maximum caliper (µm)	control	13.7	0.7	< 2e-16
**all**	morphology	cell	Maximum caliper (µm)	cisplatin	3.4	0.9	0.000
**all**	morphology	cell	Maximum caliper (µm)	docetaxel	-0.7	0.8	0.392
**all**	morphology	cell	Maximum caliper (µm)	Zn	1.2	0.9	0.165
**all**	morphology	cell	Roundness	control	0.6	0.0	<2e-16
**all**	morphology	cell	Roundness	cisplatin	0.0	0.0	0.183
**all**	morphology	cell	Roundness	docetaxel	0.0	0.0	0.107
**all**	morphology	cell	Roundness	Zn	0.0	0.0	0.917
**all**	tubulin	cell	Integrated density	control	623027.0	80495.0	0.000
**all**	tubulin	cell	Integrated density	cisplatin	29510.0	103738.0	0.776
**all**	tubulin	cell	Integrated density	docetaxel	110807.0	98695.0	0.262
**all**	tubulin	cell	Integrated density	Zn	25846.0	103738.0	0.803
**all**	tubulin	cell	Median intensity	control	178.8	10.7	< 2e-16
**all**	tubulin	cell	Median intensity	cisplatin	-32.8	13.8	0.018
**all**	tubulin	cell	Median intensity	docetaxel	69.8	13.1	0.000
**all**	tubulin	cell	Median intensity	Zn	3.3	13.8	0.812
**all**	tubulin	periphery	Integrated density	control	352467.0	26859.0	< 2e-16
**all**	tubulin	periphery	Integrated density	cisplatin	5896.0	34615.0	0.865
**all**	tubulin	periphery	Integrated density	docetaxel	100400.0	32932.0	0.002
**all**	tubulin	periphery	Integrated density	Zn	28983.0	34615.0	0.403
**all**	tubulin	periphery	Median intensity	control	174.8	11.5	< 2e-16
**all**	tubulin	periphery	Median intensity	cisplatin	-30.6	14.8	0.039
**all**	tubulin	periphery	Median intensity	docetaxel	75.0	14.1	0.000
**all**	tubulin	periphery	Median intensity	Zn	2.8	14.8	0.850
**all**	tubulin	periphery	Periphery/cell integrated density ratio	control	0.8	0.0	< 2e-16
**all**	tubulin	periphery	Periphery/cell integrated density ratio	cisplatin	-0.1	0.0	0.002
**all**	tubulin	periphery	Periphery/cell integrated density ratio	docetaxel	0.0	0.0	0.174
**all**	tubulin	periphery	Periphery/cell integrated density ratio	Zn	0.0	0.0	0.064
**22Rv1**	actin	cell	Integrated density	control	192318.0	131008.0	0.145
**22Rv1**	actin	cell	Integrated density	cisplatin	1307564.0	178279.0	0.000
**22Rv1**	actin	cell	Integrated density	docetaxel	355896.0	157336.0	0.025
**22Rv1**	actin	cell	Integrated density	Zn	310299.0	167715.0	0.067
**22Rv1**	actin	cell	Median intensity	control	149.8	20.9	0.000
**22Rv1**	actin	cell	Median intensity	cisplatin	168.0	28.4	0.000
**22Rv1**	actin	cell	Median intensity	docetaxel	76.1	25.1	0.003
**22Rv1**	actin	cell	Median intensity	Zn	73.0	26.7	0.007
**22RV1**	actin	fibers	Number of fibers	control	0.3	0.4	0.450
**22RV1**	actin	fibers	Number of fibers	cisplatin	2.3	0.5	0.000
**22RV1**	actin	fibers	Number of fibers	docetaxel	0.5	0.4	0.282
**22RV1**	actin	fibers	Number of fibers	Zn	1.1	0.5	0.019
**22Rv1**	actin	fibers	SD of angle	control	41.0	16.3	0.016
**22Rv1**	actin	fibers	SD of angle	cisplatin	-11.5	16.9	0.500

**22Rv1**	actin	fibers	SD of angle	docetaxel	-12.7	17.0	0.460
**22Rv1**	actin	fibers	SD of angle	Zn	-15.0	17.0	0.383
**22Rv1**	actin	fibers	Fiber lenght (µm)	control	4.8	1.2	0.000
**22Rv1**	actin	fibers	Fiber lenght (µm)	cisplatin	0.2	1.3	0.856
**22Rv1**	actin	fibers	Fiber lenght (µm)	docetaxel	3.6	1.3	0.008
**22Rv1**	actin	fibers	Fiber lenght (µm)	Zn	1.1	1.3	0.407
**22Rv1**	actin	fibers	Integrated density	control	326.7	307.6	0.293
**22Rv1**	actin	fibers	Integrated density	cisplatin	887.4	350.0	0.014
**22Rv1**	actin	fibers	Integrated density	docetaxel	739.2	350.0	0.040
**22Rv1**	actin	fibers	Integrated density	Zn	588.6	350.0	0.099
**22Rv1**	actin	fibers	Mean intensity	control	192.8	96.0	0.050
**22Rv1**	actin	fibers	Mean intensity	cisplatin	382.1	109.2	0.001
**22Rv1**	actin	fibers	Mean intensity	docetaxel	113.7	109.2	0.302
**22Rv1**	actin	fibers	Mean intensity	Zn	184.2	109.2	0.097
**22Rv1**	actin	periphery	Integrated density	control	182771.0	74559.0	0.016
**22Rv1**	actin	periphery	Integrated density	cisplatin	768006.0	101462.0	0.000
**22Rv1**	actin	periphery	Integrated density	docetaxel	211428.0	89542.0	0.020
**22Rv1**	actin	periphery	Integrated density	Zn	182966.0	95450.0	0.057
**22Rv1**	actin	periphery	Median intensity	control	152.6	21.0	0.000
**22Rv1**	actin	periphery	Median intensity	cisplatin	184.1	28.5	0.000
**22Rv1**	actin	periphery	Median intensity	docetaxel	81.6	25.2	0.002
**22Rv1**	actin	periphery	Median intensity	Zn	73.8	26.8	0.007
**22Rv1**	actin	periphery	Periphery/cell integrated density ratio	control	1.0	0.0	< 2e-16
**22Rv1**	actin	periphery	Periphery/cell integrated density ratio	cisplatin	-0.2	0.0	0.000
**22Rv1**	actin	periphery	Periphery/cell integrated density ratio	docetaxel	-0.2	0.0	0.000
**22Rv1**	actin	periphery	Periphery/cell integrated density ratio	Zn	-0.1	0.0	0.000
**22Rv1**	morphology	cell	Aspect ratio	control	2.2	0.2	<2e-16
**22Rv1**	morphology	cell	Aspect ratio	cisplatin	-0.1	0.3	0.639
**22Rv1**	morphology	cell	Aspect ratio	docetaxel	-0.3	0.2	0.172
**22Rv1**	morphology	cell	Aspect ratio	Zn	-0.3	0.2	0.150
**22Rv1**	morphology	cell	Cell area (μm^2)	control	178.9	69.5	0.011
**22Rv1**	morphology	cell	Cell area (μm^2)	cisplatin	519.9	94.6	0.000
**22Rv1**	morphology	cell	Cell area (μm^2)	docetaxel	196.9	83.4	0.020
**22Rv1**	morphology	cell	Cell area (μm^2)	Zn	164.8	88.9	0.066
**22Rv1**	morphology	cell	Maximum caliper (µm)	control	9.9	1.1	0.000
**22Rv1**	morphology	cell	Maximum caliper (µm)	cisplatin	7.9	1.5	0.000
**22Rv1**	morphology	cell	Maximum caliper (µm)	docetaxel	2.6	1.3	0.047
**22Rv1**	morphology	cell	Maximum caliper (µm)	Zn	1.6	1.4	0.239
**22Rv1**	morphology	cell	Roundness	control	0.5	0.0	<2e-16
**22Rv1**	morphology	cell	Roundness	cisplatin	0.0	0.1	0.538
**22Rv1**	morphology	cell	Roundness	docetaxel	0.1	0.1	0.042
**22Rv1**	morphology	cell	Roundness	Zn	0.1	0.1	0.118
**22Rv1**	tubulin	cell	Integrated density	control	126047.0	59525.0	0.036
**22Rv1**	tubulin	cell	Integrated density	cisplatin	419973.0	81003.0	0.000
**22Rv1**	tubulin	cell	Integrated density	docetaxel	321173.0	71487.0	0.000
**22Rv1**	tubulin	cell	Integrated density	Zn	244625.0	76203.0	0.002
**22Rv1**	tubulin	cell	Median intensity	control	111.3	13.3	0.000
**22Rv1**	tubulin	cell	Median intensity	cisplatin	8.6	18.1	0.635

**22Rv1**	tubulin	cell	Median intensity	docetaxel	71.3	16.0	0.000
**22Rv1**	tubulin	cell	Median intensity	Zn	50.0	17.0	0.004
**22Rv1**	tubulin	periphery	Integrated density	control	117907.0	25984.0	0.000
**22Rv1**	tubulin	periphery	Integrated density	cisplatin	190527.0	35359.0	0.000
**22Rv1**	tubulin	periphery	Integrated density	docetaxel	197143.0	31206.0	0.000
**22Rv1**	tubulin	periphery	Integrated density	Zn	139005.0	33264.0	0.000
**22Rv1**	tubulin	periphery	Median intensity	control	111.4	13.0	0.000
**22Rv1**	tubulin	periphery	Median intensity	cisplatin	8.4	17.7	0.634
**22Rv1**	tubulin	periphery	Median intensity	docetaxel	69.2	15.6	0.000
**22Rv1**	tubulin	periphery	Median intensity	Zn	47.7	16.6	0.005
**22Rv1**	tubulin	periphery	Periphery/cell integrated density ratio	control	0.9	0.0	< 2e-16
**22Rv1**	tubulin	periphery	Periphery/cell integrated density ratio	cisplatin	-0.3	0.0	0.000
**22Rv1**	tubulin	periphery	Periphery/cell integrated density ratio	docetaxel	-0.2	0.0	0.000
**22Rv1**	tubulin	periphery	Periphery/cell integrated density ratio	Zn	-0.1	0.0	0.001
**PC-3**	actin	cell	Integrated density	control	1490356.0	368906.0	0.000
**PC-3**	actin	cell	Integrated density	cisplatin	909408.0	528193.0	0.088
**PC-3**	actin	cell	Integrated density	docetaxel	44589.0	459674.0	0.923
**PC-3**	actin	cell	Integrated density	Zn	603765.0	505145.0	0.235
**PC-3**	actin	cell	Median intensity	control	307.3	29.2	< 2e-16
**PC-3**	actin	cell	Median intensity	cisplatin	-17.8	41.8	0.670
**PC-3**	actin	cell	Median intensity	docetaxel	141.7	36.4	0.000
**PC-3**	actin	cell	Median intensity	Zn	72.1	40.0	0.074
**PC-3**	actin	fibers	Number of fibers	control	2.8	0.6	0.000
**PC-3**	actin	fibers	Number of fibers	cisplatin	-0.1	0.9	0.870
**PC-3**	actin	fibers	Number of fibers	docetaxel	-0.8	0.8	0.290
**PC-3**	actin	fibers	Number of fibers	Zn	-0.8	0.8	0.318
**PC-3**	actin	fibers	SD of angle	control	19.8	4.5	0.000
**PC-3**	actin	fibers	SD of angle	cisplatin	8.7	6.6	0.193
**PC-3**	actin	fibers	SD of angle	docetaxel	7.9	6.1	0.196
**PC-3**	actin	fibers	SD of angle	Zn	9.2	6.8	0.182
**PC-3**	actin	fibers	Fiber lenght (µm)	control	4.7	0.4	0.000
**PC-3**	actin	fibers	Fiber lenght (µm)	cisplatin	0.0	0.6	0.992
**PC-3**	actin	fibers	Fiber lenght (µm)	docetaxel -	0.0	0.6	0.991
**PC-3**	actin	fibers	Fiber lenght (µm)	Zn	1.0	0.6	0.126
**PC-3**	actin	fibers	Integrated density	control	716.1	124.5	0.000
**PC-3**	actin	fibers	Integrated density	cisplatin	104.3	182.3	0.569
**PC-3**	actin	fibers	Integrated density	docetaxel	322.9	160.8	0.049
**PC-3**	actin	fibers	Integrated density	Zn	379.8	182.3	0.041
**PC-3**	actin	fibers	Mean intensity	control	388.3	50.2	0.000
**PC-3**	actin	fibers	Mean intensity	cisplatin	61.1	73.5	0.409
**PC-3**	actin	fibers	Mean intensity	docetaxel	155.6	64.8	0.019
**PC-3**	actin	fibers	Mean intensity	Zn	101.7	73.5	0.171
**PC-3**	actin	periphery	Integrated density	control	791526.0	110027.0	0.000
**PC-3**	actin	periphery	Integrated density	cisplatin	284031.0	157535.0	0.074
**PC-3**	actin	periphery	Integrated density	docetaxel	144596.0	137099.0	0.294
**PC-3**	actin	periphery	Integrated density	Zn	291084.0	150661.0	0.056
**PC-3**	actin	periphery	Median intensity	control	329.4	32.0	< 2e-16
**PC-3**	actin	periphery	Median intensity	cisplatin	-9.8	45.9	0.831

**PC-3**	actin	periphery	Median intensity	docetaxel	196.8	39.9	0.000
**PC-3**	actin	periphery	Median intensity	Zn	101.9	43.9	0.022
**PC-3**	actin	periphery	Periphery/cell integrated density ratio	control	0.6	0.0	<2e-16
**PC-3**	actin	periphery	Periphery/cell integrated density ratio	cisplatin	-0.1	0.1	0.014
**PC-3**	actin	periphery	Periphery/cell integrated density ratio	docetaxel	0.1	0.0	0.046
**PC-3**	actin	periphery	Periphery/cell integrated density ratio	Zn	0.0	0.0	0.793
**PC-3**	morphology	cell	Aspect ratio	control	2.0	0.2	< 2e-16
**PC-3**	morphology	cell	Aspect ratio	cisplatin	-0.2	0.3	0.548
**PC-3**	morphology	cell	Aspect ratio	docetaxel	-0.6	0.2	0.006
**PC-3**	morphology	cell	Aspect ratio	Zn	-0.4	0.2	0.099
**PC-3**	morphology	cell	Cell area (μm^2)	control	866.7	160.4	0.000
**PC-3**	morphology	cell	Cell area (μm^2)	cisplatin	439.0	229.7	0.059
**PC-3**	morphology	cell	Cell area (μm^2)	docetaxel	-358.5	199.9	0.076
**PC-3**	morphology	cell	Cell area (μm^2)	Zn	-7.1	219.7	0.974
**PC-3**	morphology	cell	Maximum caliper (µm)	control	17.7	1.3	< 2e-16
**PC-3**	morphology	cell	Maximum caliper (µm)	cisplatin	4.0	1.9	0.039
**PC-3**	morphology	cell	Maximum caliper (µm)	docetaxel	-5.8	1.7	0.001
**PC-3**	morphology	cell	Maximum caliper (µm)	Zn	-1.4	1.8	0.437
**PC-3**	morphology	cell	Roundness	control	0.6	0.0	< 2e-16
**PC-3**	morphology	cell	Roundness	cisplatin	0.0	0.1	0.786
**PC-3**	morphology	cell	Roundness	docetaxel	0.1	0.0	0.008
**PC-3**	morphology	cell	Roundness	Zn	0.1	0.1	0.168
**PC-3**	tubulin	cell	Integrated density	control	1220513.0	245560.0	0.000
**PC-3**	tubulin	cell	Integrated density	cisplatin	306931.0	351588.0	0.385
**PC-3**	tubulin	cell	Integrated density	docetaxel	178744.0	305979.0	0.560
**PC-3**	tubulin	cell	Integrated density	Zn	7317.0	336246.0	0.983
**PC-3**	tubulin	cell	Median intensity	control	208.2	29.8	0.000
**PC-3**	tubulin	cell	Median intensity	cisplatin	-18.3	42.6	0.668
**PC-3**	tubulin	cell	Median intensity	docetaxel	202.5	37.1	0.000
**PC-3**	tubulin	cell	Median intensity	Zn	21.8	40.8	0.594
**PC-3**	tubulin	periphery	Integrated density	control	551481.0	60615.0	0.000
**PC-3**	tubulin	periphery	Integrated density	cisplatin	68363.0	86788.0	0.433
**PC-3**	tubulin	periphery	Integrated density	docetaxel	188511.0	75530.0	0.014
**PC-3**	tubulin	periphery	Integrated density	Zn	21700.0	83001.0	0.794
**PC-3**	tubulin	periphery	Median intensity	control	208.2	29.8	0.000
**PC-3**	tubulin	periphery	Median intensity	cisplatin	-18.3	42.6	0.668
**PC-3**	tubulin	periphery	Median intensity	docetaxel	202.5	37.1	0.000
**PC-3**	tubulin	periphery	Median intensity	Zn	21.8	40.8	0.594
**PC-3**	tubulin	periphery	Periphery/cell integrated density ratio	control	0.6	0.0	<2e-16
**PC-3**	tubulin	periphery	Periphery/cell integrated density ratio	cisplatin	-0.1	0.1	0.040
**PC-3**	tubulin	periphery	Periphery/cell integrated density ratio	docetaxel	0.0	0.1	0.386
**PC-3**	tubulin	periphery	Periphery/cell integrated density ratio	Zn	0.0	0.1	0.519
**PNT1A**	actin	cell	Integrated density	control	1007319.0	94594.0	<2e-16
**PNT1A**	actin	cell	Integrated density	cisplatin	-90165.0	114712.0	0.433
**PNT1A**	actin	cell	Integrated density	docetaxel	54353.0	116799.0	0.642
**PNT1A**	actin	cell	Integrated density	Zn	-25196.0	118971.0	0.832
**PNT1A**	actin	cell	Median intensity	control	399.1	19.4	< 2e-16
**PNT1A**	actin	cell	Median intensity	cisplatin	-91.3	23.6	0.000

**PNT1A**	actin	cell	Median intensity	docetaxel	0.5	24.0	0.984
**PNT1A**	actin	cell	Median intensity	Zn	-85.7	24.4	0.001
**PNT1A**	actin	fibers	Number of fibers	control	2.7	0.5	0.000
**PNT1A**	actin	fibers	Number of fibers	cisplatin	2.4	0.6	0.000
**PNT1A**	actin	fibers	Number of fibers	docetaxel	-0.6	0.6	0.294
**PNT1A**	actin	fibers	Number of fibers	Zn	0.5	0.6	0.388
**PNT1A**	actin	fibers	SD of angle	control	21.1	3.1	0.000
**PNT1A**	actin	fibers	SD of angle	cisplatin	0.6	3.6	0.877
**PNT1A**	actin	fibers	SD of angle	docetaxel	-4.4	3.8	0.247
**PNT1A**	actin	fibers	SD of angle	Zn	-4.0	3.6	0.275
**PNT1A**	actin	fibers	Fiber lenght (µm)	control	4.6	0.7	0.000
**PNT1A**	actin	fibers	Fiber lenght (µm)	cisplatin	3.0	0.8	0.000
**PNT1A**	actin	fibers	Fiber lenght (µm)	docetaxel	4.9	0.8	0.000
**PNT1A**	actin	fibers	Fiber lenght (µm)	Zn	5.0	0.8	0.000
**PNT1A**	actin	fibers	Integrated density	control	840.8	192.2	0.000
**PNT1A**	actin	fibers	Integrated density	cisplatin	281.6	227.1	0.217
**PNT1A**	actin	fibers	Integrated density	docetaxel	1372.1	233.6	0.000
**PNT1A**	actin	fibers	Integrated density	Zn	765.7	230.4	0.001
**PNT1A**	actin	fibers	Mean intensity	control	459.0	32.1	< 2e-16
**PNT1A**	actin	fibers	Mean intensity	cisplatin	-87.3	37.9	0.023
**PNT1A**	actin	fibers	Mean intensity	docetaxel	133.4	39.0	0.001
**PNT1A**	actin	fibers	Mean intensity	Zn	-41.9	38.5	0.277
**PNT1A**	actin	periphery	Integrated density	control	765959.0	59110.0	<2e-16
**PNT1A**	actin	periphery	Integrated density	cisplatin	-64400.0	71682.0	0.370
**PNT1A**	actin	periphery	Integrated density	docetaxel	40700.0	72986.0	0.578
**PNT1A**	actin	periphery	Integrated density	Zn	-36874.0	74343.0	0.620
**PNT1A**	actin	periphery	Median intensity	control	399.1	19.4	< 2e-16
**PNT1A**	actin	periphery	Median intensity	cisplatin	-91.3	23.6	0.000
**PNT1A**	actin	periphery	Median intensity	docetaxel	0.5	24.0	0.984
**PNT1A**	actin	periphery	Median intensity	Zn	-85.7	24.4	0.001
**PNT1A**	actin	periphery	Periphery/cell integrated density ratio	control	0.8	0.0	<2e-16
**PNT1A**	actin	periphery	Periphery/cell integrated density ratio	cisplatin	0.0	0.0	0.828
**PNT1A**	actin	periphery	Periphery/cell integrated density ratio	docetaxel	0.0	0.0	0.568
**PNT1A**	actin	periphery	Periphery/cell integrated density ratio	Zn	0.0	0.0	0.712
**PNT1A**	morphology	cell	Aspect ratio	control	2.0	0.2	<2e-16
**PNT1A**	morphology	cell	Aspect ratio	cisplatin	0.4	0.2	0.091
**PNT1A**	morphology	cell	Aspect ratio	docetaxel	0.2	0.2	0.502
**PNT1A**	morphology	cell	Aspect ratio	Zn	0.5	0.2	0.029
**PNT1A**	morphology	cell	Cell area (μm^2)	control	400.9	42.4	<2e-16
**PNT1A**	morphology	cell	Cell area (μm^2)	cisplatin	40.6	51.4	0.431
**PNT1A**	morphology	cell	Cell area (μm^2)	docetaxel	20.0	52.3	0.702
**PNT1A**	morphology	cell	Cell area (μm^2)	Zn	67.4	53.3	0.207
**PNT1A**	morphology	cell	Maximum caliper (µm)	control	13.8	1.0	<2e-16
**PNT1A**	morphology	cell	Maximum caliper (µm)	cisplatin	1.7	1.2	0.159
**PNT1A**	morphology	cell	Maximum caliper (µm)	docetaxel	0.3	1.2	0.811
**PNT1A**	morphology	cell	Maximum caliper (µm)	Zn	2.7	1.2	0.028
**PNT1A**	morphology	cell	Roundness	control	0.6	0.0	<2e-16
**PNT1A**	morphology	cell	Roundness	cisplatin	-0.1	0.0	0.036

**PNT1A**	morphology	cell	Roundness	docetaxel	0.0	0.0	0.276
**PNT1A**	morphology	cell	Roundness	Zn	-0.1	0.0	0.043
**PNT1A**	tubulin	cell	Integrated density	control	588131.0	55432.0	<2e-16
**PNT1A**	tubulin	cell	Integrated density	cisplatin	-150627.0	67222.0	0.026
**PNT1A**	tubulin	cell	Integrated density	docetaxel	-24497.0	68445.0	0.721
**PNT1A**	tubulin	cell	Integrated density	Zn	-9799.0	69718.0	0.888
**PNT1A**	tubulin	cell	Median intensity	control	208.6	8.9	< 2e-16
**PNT1A**	tubulin	cell	Median intensity	cisplatin	-65.2	10.8	0.000
**PNT1A**	tubulin	cell	Median intensity	docetaxel	-0.3	11.0	0.976
**PNT1A**	tubulin	cell	Median intensity	Zn	-34.5	11.3	0.002
**PNT1A**	tubulin	periphery	Integrated density	control	390453.0	28542.0	<2e-16
**PNT1A**	tubulin	periphery	Integrated density	cisplatin	-89172.0	34612.0	0.011
**PNT1A**	tubulin	periphery	Integrated density	docetaxel	1031.0	35242.0	0.977
**PNT1A**	tubulin	periphery	Integrated density	Zn	-11153.0	35897.0	0.756
**PNT1A**	tubulin	periphery	Median intensity	control	198.5	9.1	< 2e-16
**PNT1A**	tubulin	periphery	Median intensity	cisplatin	-58.1	11.0	0.000
**PNT1A**	tubulin	periphery	Median intensity	docetaxel	10.0	11.2	0.373
**PNT1A**	tubulin	periphery	Median intensity	Zn	-31.6	11.5	0.006
**PNT1A**	tubulin	periphery	Periphery/cell integrated density ratio	control	0.7	0.0	<2e-16
**PNT1A**	tubulin	periphery	Periphery/cell integrated density ratio	cisplatin	0.0	0.0	0.925
**PNT1A**	tubulin	periphery	Periphery/cell integrated density ratio	docetaxel	0.0	0.0	0.861
**PNT1A**	tubulin	periphery	Periphery/cell integrated density ratio	Zn	0.0	0.0	0.688
